# The Detection of DNA Damage Response in MCF7 and MDA-MB-231 Breast Cancer Cell Lines after X-ray Exposure

**DOI:** 10.14293/genint.14.1.001

**Published:** 2023-01-04

**Authors:** Alkhansa Mahmoud, Arianna Casciati, Zuki Abu Bakar, Hazilawati Hamzah, Tengku Ahbrizal Tengku Ahmad, Mohd Hezmee Mohd Noor

**Affiliations:** 1 Pharmacology and Toxicology Laboratory, Faculty of Veterinary Medicine, University Putra Malaysia, 43400 Serdang, Selangor, Malaysia; 2 Radiobiology Department, Sudan Atomic Energy Commission, 11111 Khartoum, Sudan; 3 Biomedical Technologies Laboratory, Italian National Agency for New Technologies, Energy and Sustainable Economic Development (ENEA), 00123 Rome, Italy; 4 Department of Veterinary Preclinical Sciences, Faculty of Veterinary Medicine, University Putra Malaysia, 43400 Serdang, Selangor, Malaysia; 5 Department of Veterinary Pathology and Microbiology, Faculty of Veterinary Medicine, University Putra Malaysia, 43400 Serdang, Selangor, Malaysia; 6 Radiation Health and Safety Division, Malaysian Nuclear Agency, Bangi, 43000 Kajang, Selangor, Malaysia

**Keywords:** Breast cancer, cell lines, X-ray, radiation

## Abstract

Radiotherapy is one of the main options to cure and control breast cancer. The aim of this study was to investigate the sensitivity of two human breast cancer cell lines, MCF7 and MDA-MD-231, to radiation exposure at timepoints 4 h and 24 h after radiation. MCF7 and MDA-MD-231 were irradiated with different radiation doses using a Gilardoni CHF 320 G X-ray generator (Mandello del Lario, Italy) at 250 kVp, 15 mA [with half-value layer (HVL) = 1.6 mm copper]. The ApoTox-Glo triplex assay combines three assays used to assess viability, cytotoxicity, and apoptosis. The expression of γH2AX and BAX was analyzed by Western blotting. Viability and cytotoxicity did not change 4 h and 24 h after irradiation in either cell line, but we found a significant increase in the expression of cleaved caspase-3/7 at 24 h after irradiation with 8.5 Gy in MDA-MB231. The expression of γH2AX and BAX was low in MCF7, whereas the expression of γH2AX and BAX increased with radiation dose in a dose-dependent manner in MDA-MB231. The results show that the MCF7 cell line is more radioresistant than the MDA-MB 231 cell line at 4 h and 24 h after X-ray irradiation. In contrast, MDA-MB-231 cells were radiosensitive at a high radiation dose of 8.5 Gy at 24 h after irradiation. γH2AX and BAX indicated the radiosensitivity in both cell lines. These results open the possibility of using these cancer cell lines as models for testing new therapeutic strategies to improve radiation therapy.

## Introduction

Breast cancer is the most common cause of death due to cancer in women worldwide.^[1]^ Breast cancer is classified into different subgroups depending on whether it expresses estrogen receptor (ER), progesterone receptor (PR), or human epidermal growth factor receptor 2 (HER2).^[2]^ Radiotherapy is one of the most important options for the cure and control of breast cancer after surgery;^[3]^ more than 83% of breast cancer patients benefit from radiotherapy, either curative or palliative.^[4]^ Radiation has significantly reduced the risk of local recurrence and also improved overall survival.^[5]^ However, cancer cells may acquire radioresistance, which is associated with an increased risk of death.^[6]^ In addition, the discovery of targets that predict response to radiotherapy and agents that sensitize cancer cells to ionizing radiation with minimal side effects is of great interest. However, in certain subtypes of breast cancer (e.g., basal-like), local and regional control remains unsatisfactory.^[7]^ A major reason for this treatment failure could be radiation resistance. Therefore, understanding the molecular mechanisms involved in radiation resistance of breast tumors could lead to better clinical outcomes.^[8]^ Radiation is a physical agent used to destroy cancer cells as it forms ions and deposits energy in the cells of the tissue it penetrates. This deposited energy can kill cancer cells or cause genetic changes that lead to cancer cell death.^[9]^ Ionizing radiation is one of the main treatment options for all stages of breast cancer.^[10]^ Radiation therapy exerts its effects by causing DNA damage either directly or indirectly through the production of water-derived radicals and reactive oxygen species,^[11]^ which then interact with macromolecules such as DNA, lipids, and proteins. The DNA damage response (DDR) is triggered, leading to the activation of DNA damage repair mechanisms and the induction of checkpoint kinase pathways for DNA repair, which induces apoptosis in cancer cells.^[12]^ Phosphorylation of histone H2AX at serine 139 (γH2AX) is considered a sensitive marker for ionizing radiation-induced double strand breaks (DSBs); γH2AX plays a functional and structural role in DNA damage and repair.^[13]^ Thus, γH2AX may act as a sensitive marker for DSBs, which in turn may signify genomic instability and contribute to cancer development and progression, and is considered an important regulator of DNA damage and repair.^[14]^ The MCF7 cell has been widely used as a good model for ER-positive breast tumors, while the MDA-MB231 cell lines, which do not express ER, are used as a model for studying ER-negative breast tumors. However, tumor radioresistance remains the main problem in the efficacy of radiotherapy for the treatment of breast cancer. Radioresistance may be present at the beginning of therapy and cause patients not to respond to therapy, or cancer cells may develop radioresistance during radiotherapy, leading to treatment failure.^[15]^ As radioresistance is the main reason for treatment failure, further studies to understand the response of tumor cells exposed to radiation are needed to improve the effect of radiotherapy. The aim of this study is to investigate the radiosensitivity of MCF7 and MDA-MB-231 cell lines using an X-ray irradiator with different radiation doses at 4 h and 24 h after irradiation. These two time points have been selected because apoptosis begin within several hours after irradiation.^[16]^

## Materials and Methods

### Cell culture

MCF7 and MDA-MB-231 cell lines were thawed from liquid nitrogen, washed and cultured in Dulbecco’s Modified Eagle medium (DMEM-F12) supplemented with 10% fetal bovine serum (FBS), penicillin/streptomycin, and glutamine 37°C humidified 5% carbon dioxide (CO_2_) atmosphere.

### Cell irradiation

MCF7 and MDA-MB-231 cells were plated in T75 flasks and then irradiated using a Gilardoni CHF 320 G X-ray generator (Mandello del Lario, Italy) operating at 250 kV, 15 mA [with half-value layer (HVL) = 1.6 mm copper] with various radiation doses (2 Gy, 5 Gy, and 8.5 Gy) with a dose rate of 0.89 Gy/min at temperature 20°C. The control cells (0 Gy) were treated the same as the radiation cells.

### The ApoTox-Glo™ Triplex Assay (Promega)

The ApoTox-Glo™ Triplex Assay (Promega, Madison, Wisconsin, USA) assay measures cell viability, cytotoxicity, and, apoptosis in one assay, it was used according to the manufacturer’s protocol, and the reagents prepared as follows: each assay component was thawed and included each assay buffer: a 37°C water bath, GF-AFC substrate in a 37°C water bath, bis-AAF-R110 substrate in a 37°C water bath, Caspase-Glo^®^ 3/7 Buffer at room temperature and Caspase-Glo^®^ 3/7 substrate at room temperature.

For the viability/cytotoxicity reagent, the contents of the GF-AFC substrate and bis-AAF-R110 substrate were transferred into 2.0 ml of assay buffer, depending on the plate format used. For 96-well plates, 10 μl of each substrate was transferred into 2 ml of assay buffer, and the assay buffer containing substrates was mixed using a vortex until the substrates were thoroughly dissolved. For the apoptosis assay, the contents of the Caspase-Glo^®^ 3/7 buffer bottle were transferred into an amber bottle containing Caspase-Glo^®^ 3/7 substrate, and the contents were mixed by swirling until the substrate was thoroughly dissolved to form the Caspase-Glo^®^ 3/7 reagent (~20 s).

Then, the assay protocol for a 96-well plate format was performed by setting up 96-well assay plates containing cells in medium at the selected density (10,000 cells), and test compounds and vehicle controls were added to the appropriate wells for a final volume of 100 μl/well. The cultured cells were exposed for the desired test period (4 h and 24 h). A total of 20 μl of viability/cytotoxicity reagent containing both GF-AFC substrate and bis-AAF-R110 substrate were added to all the wells, and were briefly mixed using orbital shaking (300–500 rpm for ~30 s) and incubated for 30 min at 37°C. One plate was read at 4 h and one plate was read at 24 h. A second reagent containing luminogenic DEVD-peptide substrate for caspase-3/7 and Ultra-Glo™ Recombinant Thermostable Luciferase were also added. Caspase-3/7 cleavage of the substrate releases luciferin, which is a substrate for luciferase and generates light. The light output, measured with a luminometer, correlates with Caspase-3/7 activation as a key indicator of apoptosis.

### Western blotting protocol

To assess the differential cytotoxicity of X-rays, we analyzed γH2AX for DSBs and Bax for apoptosis in MCF7 and MDA-MB-231 cell lines using the Western blot technique after exposure of 2 Gy, 5Gy, and 8.5Gy. After the cells were irradiated they were incubated in a CO_2_ incubator for 30 min before γH2AX was analyzed because this marker reached maximum levels at 30 min after irradiation,^[17]^ and 4 h in a CO_2_ incubator after irradiation for Bax (apoptosis regulator BAX). The Western blotting protocol was performed as follows: the cells were lysed and Bradford protein assay was used for as a protein assay. Primary antibodies γH2AX (cat no. 2577, Cell Signalling) and Bax (cat no.2772, Cell Signalling) were diluted 1:1000 in a blocking buffer and incubated at 4°C overnight in a slow shaker. The primary antibodies were then removed and the membranes were washed three times with tris-buffered saline + Tween 20 (TBST) and shaken for 10 min at room temperature in a fast shaker. A secondary antibody was added (anti-rabbit) horseradish peroxidase (HRP) (Santa Cruz Biotechnology, Santa Cruz, TX, USA) and diluted 1:2000 in blocking buffer for 1 h in a slow shaker at room temperature. The secondary antibody was removed and washed three times with TBST for 10 min on a slow shaker at room temperature. The housekeeping gene β-actin (Human/Mouse/Rat beta – Actin Antibody, MAB8929) was used for normalization, as the same protocol as mentioned above was used for the targeted proteins γH2AX and BAX. Proteins were electro transferred to polyvinylidene difluoride membranes (Trans-Blot Turbo Transfer Pack, Bio-Rad Laboratories, Hercules, CA, USA) in a blotting machine (Bio-Rad). Immunoreactive bands were visualized using Amersham ECL Prime WB detection reagent (GE Healthcare Europe, Milan, Italy). Images were acquired using Image 6 quant LAS 500 (GE Healthcare Europe), and densitometric analysis was performed using ImageJ software (Corning).

### Statistical analysis

Data are presented as the means ± standard deviation and analyzed using Student’s *t*-test. Statistical analyses were performed using GraphPad Prism software (USA). A *P*-value of < 0.05 was considered to be statistically significant.^[18]^

## Results

**Figure 1 (A, B):**
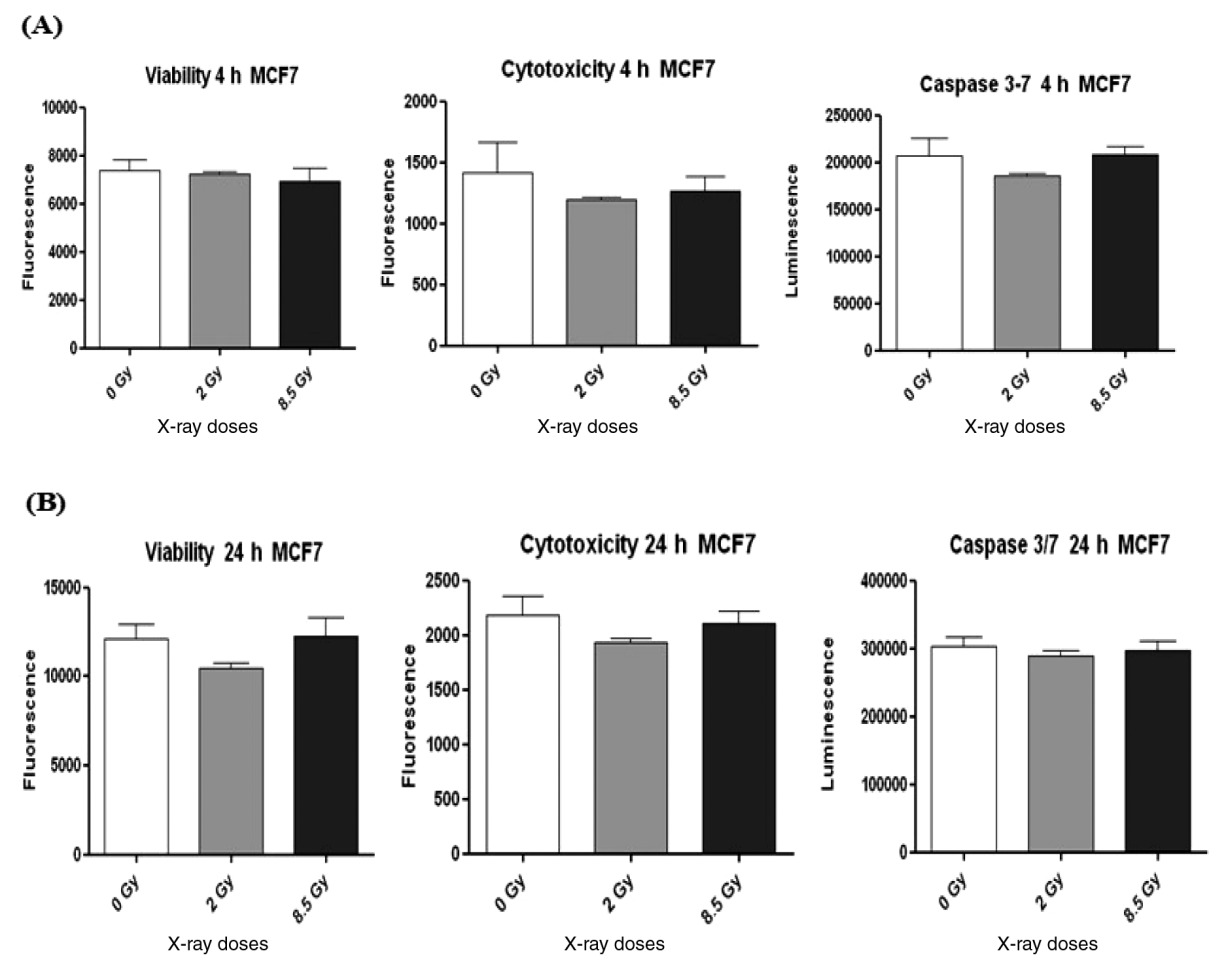
ApoTox-Glo Triplex Assay in the MCF7 cell line at 4 h and 24 h after X-ray irradiation.

**Figure 2 (A, B):**
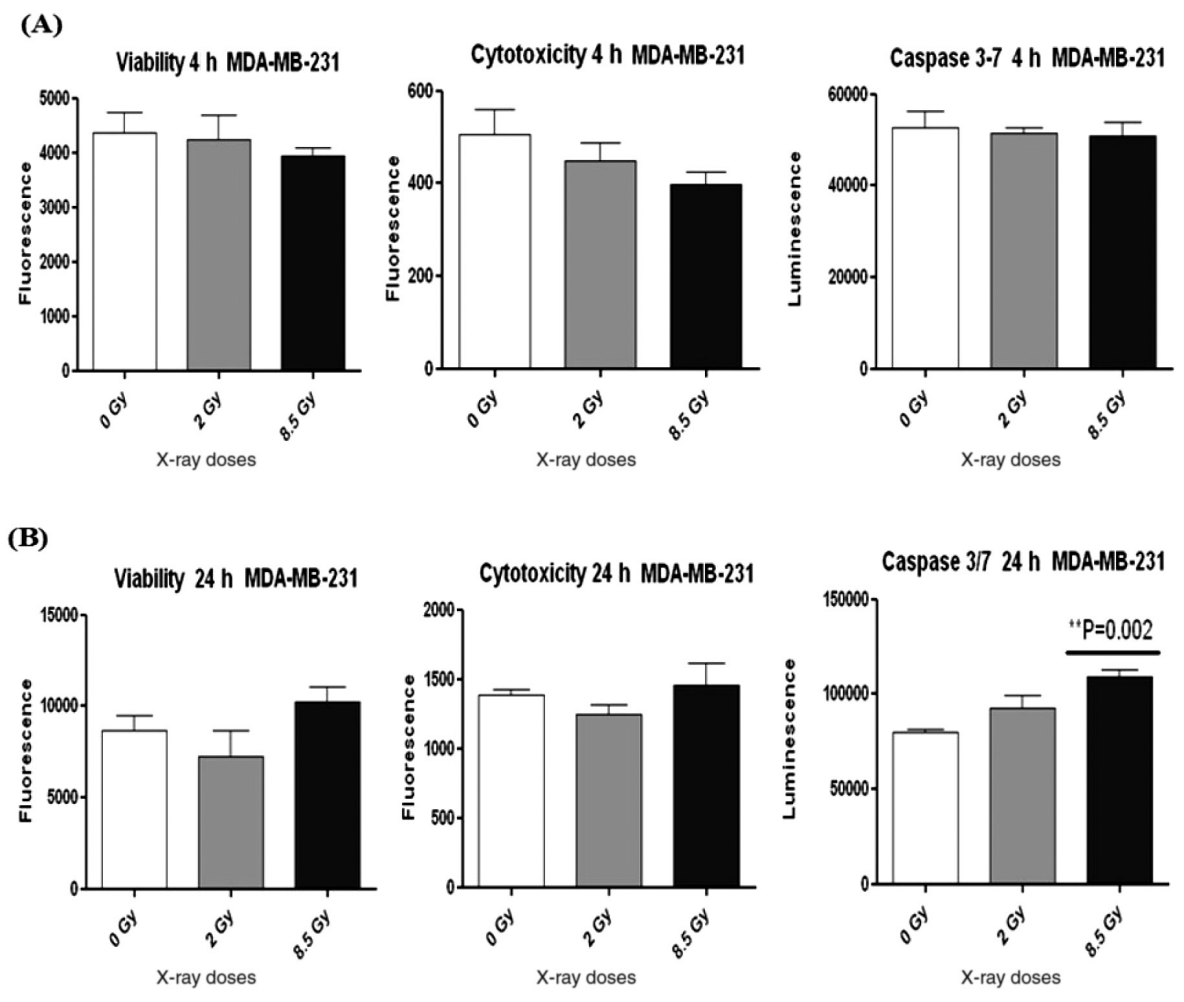
ApoTox-Glo Triplex Assay in the MDA-MB-231 Cell line at 4 h and 24 h after X-ray irradiation.

**Figure 3:**
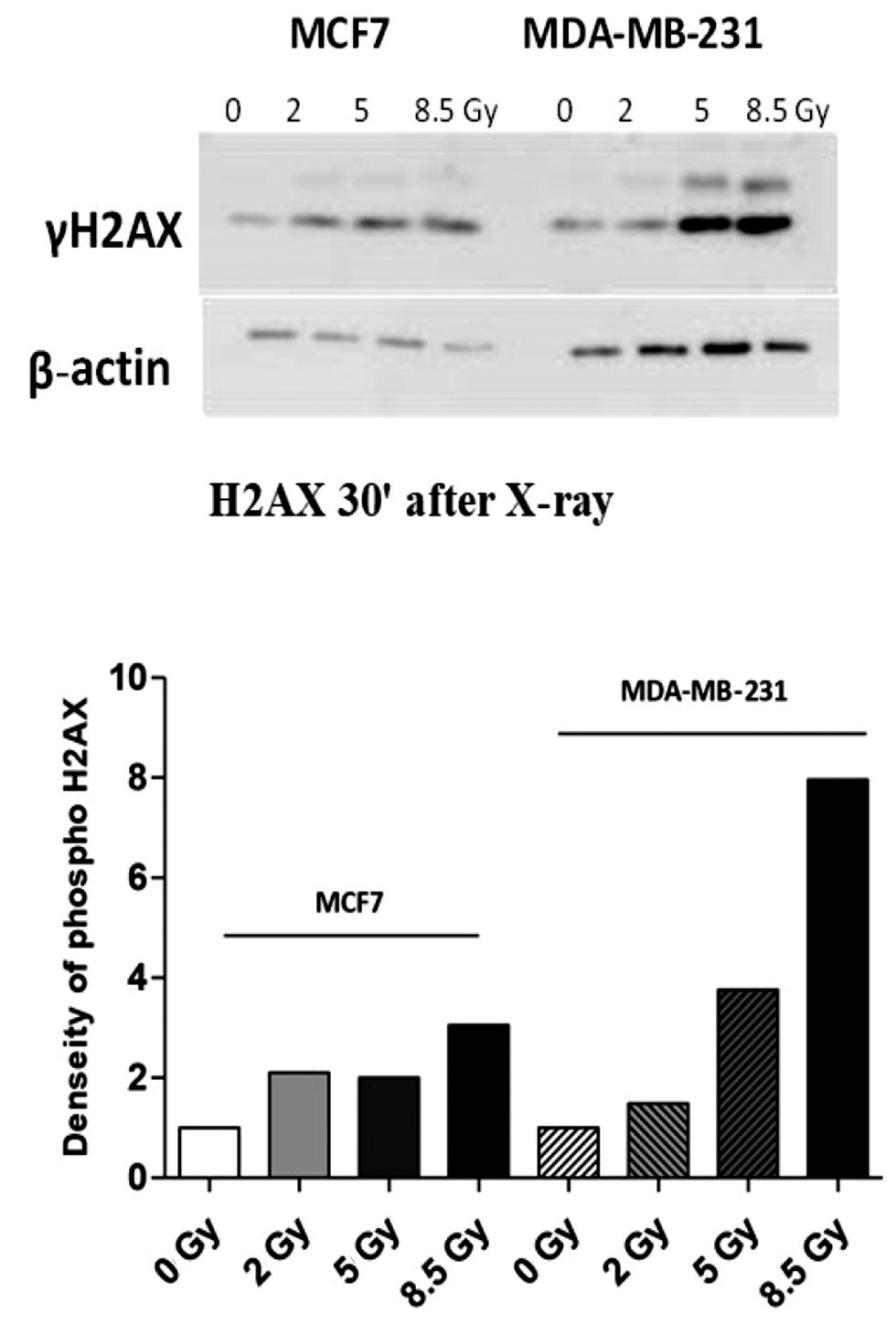
Effects of X-rays on the expression level of γH2AX in MCF7 and MDA-MB-231 cells. The expression levels of the DNA DSB marker γH2AX in MCF7 and MDA-MB-231 cells measured by Western blotting assay 30 min after X-ray irradiation with doses of 0, 2, 5, and 8.5 Gy.

**Figure 4:**
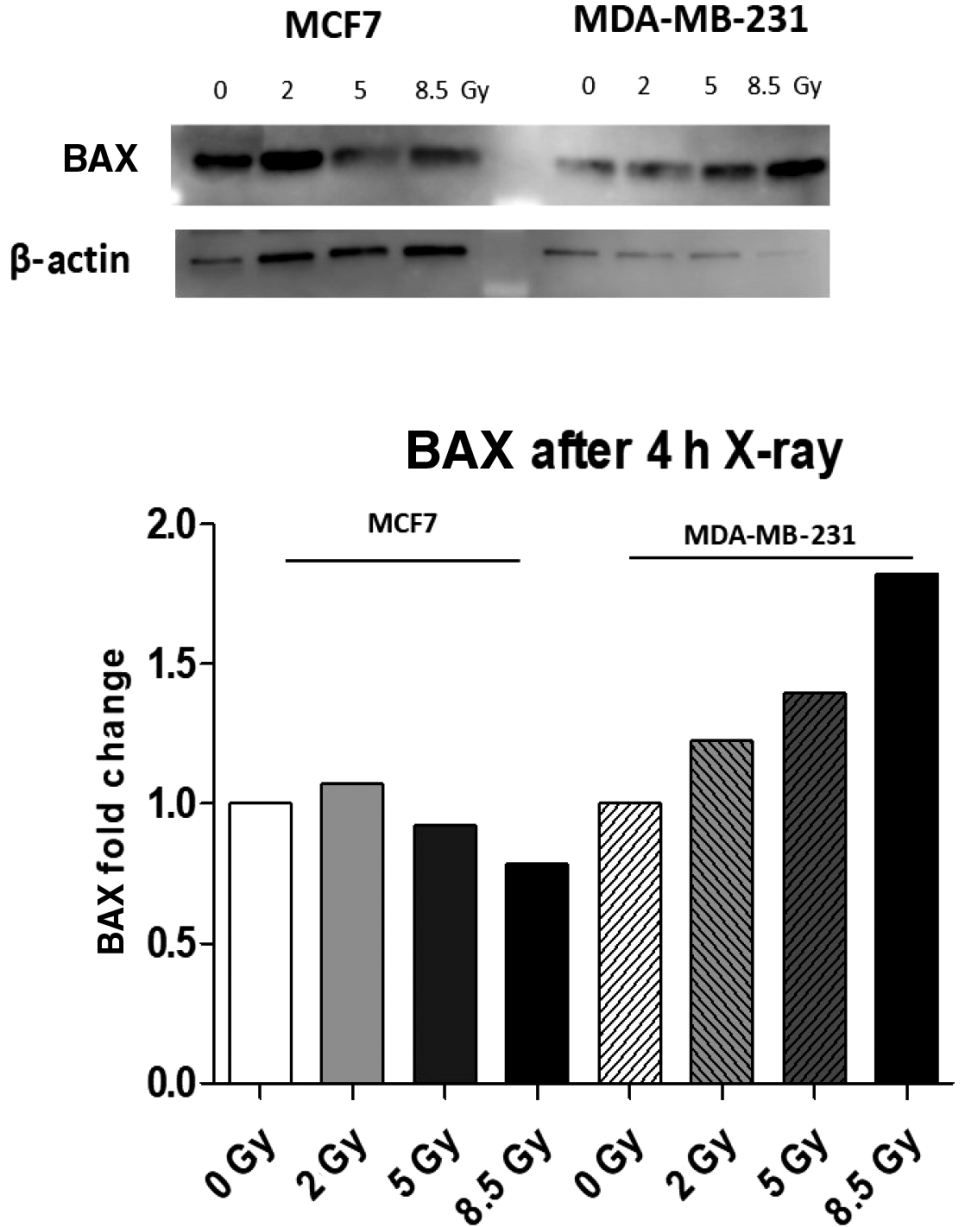
Effects of X-rays on the expression of BAX in MCF7 and MDA-MB-231 cells. It showed the expression levels of BAX in MCF7 and MDA-MB-231 cells measured 4 h after X-ray irradiation with doses of 0, 2, 5 and 8.5 Gy by Western blotting assay.

MCF7 cells did not show changes in viability, cytotoxicity, and apoptosis at 4 h and 24 h after irradiation as shown in Figure 1 (A, B). However, MCF7 cells at 24 h showed a decrease in viability and cytotoxicity without action in Caspase 3/7 that appears to show that apoptosis has not started yet as shown in Figure 1(B). Regarding the MDA-MB-231 cell line, there was no significant difference in viability, cytotoxicity, and apoptosis at 4 h as in Figure 2 (A). In contrast, a significant increase in Caspase 3/7 expression was detected at 24 h after irradiation with 8.5 Gy (*P* = 0.002), as shown in Figure 2 (B). Meanwhile, 2 Gy at 24 h showed a decrease in viability and cytotoxicity with increased in Caspase 3/7 expression, but no significant different was observed (Figure 2B), thus it appears that MDA-MB-231 cells need to improve radiosensitivity at 2 Gy. The expression of γH2AX and BAX were low in MCF7, whereas the expression of γH2AX and BAX increased in a dose-dependent manner in the MDA-MB-231 cell line, as shown in Figures 3 and 4, respectively.

## Discussion

Radiation therapy is usually a balancing act between delivering enough dose to achieve local tumor control and only as much dose as the surrounding tissue can tolerate. But not all cancer cells respond to radiation therapy. Therefore, it is very important to understand the different radiation sensitivity of different tumor cells. Inadequate response to radiation or resistance to radiation contributes to the remaining cancer mass, which is the key to recurrence.^[19]^

The stem cell model of cancer development can explain genetic, functional, and phenotypic differences, such as increased resistance to therapy including radiotherapy.^[20]^ Therefore, a detailed understanding of the differential sensitivity of cancer cells, especially cancer stem cells (CSC), to radiation is crucial as it may help to understand the characterization of the cells to develop new or improved cancer therapies.^[21]^ Previous studies that demonstrated the radiosensitivity of CSC have been performed in breast cancer and glioma.^[22]^ MCF7 and MDA-MB-231 cells are known documented differences between the two types. They have many phenotypic and genotypic differences. MCF7 cells are hormone-dependent, and estrogen and progesterone receptor positive (ER + and PR +), while MDA-MB-231 cells are a triple negative, i.e., they lack estrogen and progesterone receptors and also hormone epidermal growth factor 2 (HER2). MDA-MB-231 cells are more aggressive and have a worse prognosis. In the present study, MCF7 and MDA-MB-231 were analyzed based on their response to X-ray irradiation to evaluate cell viability, cytotoxicity, and apoptosis, as well as DNA DSBs. MCF7 and MDA-MB-231 were used due to their different characteristics. However, understanding the extent of DNA breakage is particularly important for the study of tumorigenesis because many cancers are known to have mutations in the DNA damage response pathways responsible for repairing DSBs which contribute to genomic instability that leads to tumor development. In general, cancer is characterized by increasing cell growth, decreasing apoptosis, and inhibiting programmed cell death.^[23]^ A previous study confirmed our findings and reported that MCF7 exhibited greater radioresistance than the MDA-MB-231 cell line.^[24]^ Some studies found that the radiosensitivity of the ER-negative breast cancer cell line MDA-MB-231 was significantly lower than that of the ER-positive breast cancer MCF7 cell line,^[25]^ which is in contrast to our results showing that the MDA-MB-231 cell lines are more radiosensitive than the MCF7 cell line. In the MDA-MB-231 cell line, the expression of BAX and γH2AX increased sharply with increasing radiation dose. MCF7 showed no difference in cytotoxicity and apoptosis compared with the control. The expression levels of γH2AX and BAX also increased only slightly after irradiation in MCF7. Another study confirmed our results and reported that MDA-MB-231 cells were not radiosensitive at a dose of 2 Gy.^[26]^ Although ionizing radiation induces cell death in MDA-MB-231 cells at a dose of 8.5 Gy, the response at a high dose is also considered a poor response because such a high dose of radiation is required to achieve results. These results show that the MDA-MB-231 cell line is more aggressive and has a poorer prognosis, while MCF7 is radioresistant at both low and high doses, implying that these cells require enhancement of the radiation response, such as enhancing the cells for radiation with a radiosensitizer. Also, the radiation response in MCF7 was achieved by 24 h after irradiation, however, time points are important in radiosensitivity monitoring, some of cancer cells begin apoptosis by mechanisms that occurred after 72 h.^[27]^ Our study focused on early apoptosis based on 4 h and 24 h. Furthermore, γH2AX and BAX biomarkers can reflect the radiosensitivity status in both cell lines.

## Conclusion

The results showed that viability and cytotoxicity do not change significantly at 24 h after exposure in both cell lines, whereas a significant increase in cleaved Caspase 3/7 was detected in MDA-MB-231 at 24 h after 8.5 Gy irradiation. The results suggest that the MCF7 cell line is more resistant to X-ray irradiation than the MDA-MB-231 cell line. Furthermore, the results suggest the possibility of using these cancer cell lines as models for testing new therapeutic strategies to improve radiotherapy.
